# Reconstruction of High-Precision Semantic Map

**DOI:** 10.3390/s20216264

**Published:** 2020-11-03

**Authors:** Xinyuan Tu, Jian Zhang, Runhao Luo, Kai Wang, Qingji Zeng, Yu Zhou, Yao Yu, Sidan Du

**Affiliations:** School of Electronic Science and Engineer, Nanjing University, Nanjing 21000, China; MG20230019@smail.nju.edu.cn (X.T.); jianzhang@smail.nju.edu.cn (J.Z.); mf1723032@smail.nju.edu.cn (R.L.); mf1723048@smail.nju.edu.cn (K.W.); 151180009@smail.nju.edu.cn (Q.Z.); allanyu@nju.edu.cn (Y.Y.); coff128@nju.edu.cn (S.D.)

**Keywords:** 3D semantic map, 3D reconstruction, semantic fusion

## Abstract

We present a real-time Truncated Signed Distance Field (TSDF)-based three-dimensional (3D) semantic reconstruction for LiDAR point cloud, which achieves incremental surface reconstruction and highly accurate semantic segmentation. The high-precise 3D semantic reconstruction in real time on LiDAR data is important but challenging. Lighting Detection and Ranging (LiDAR) data with high accuracy is massive for 3D reconstruction. We so propose a line-of-sight algorithm to update implicit surface incrementally. Meanwhile, in order to use more semantic information effectively, an online attention-based spatial and temporal feature fusion method is proposed, which is well integrated into the reconstruction system. We implement parallel computation in the reconstruction and semantic fusion process, which achieves real-time performance. We demonstrate our approach on the CARLA dataset, Apollo dataset, and our dataset. When compared with the state-of-art mapping methods, our method has a great advantage in terms of both quality and speed, which meets the needs of robotic mapping and navigation.

## 1. Introduction

When entering unfamiliar environment, it is very important to perceive the 3D structure and semantic information in real time. Reconstructing precise and continuous surface in real time allows for robots to respond accurately and fast. At the same time, fusing semantic information into the 3D map is also a necessary part from the perception standpoint. In this work, we are interested in online feature fusion and 3D reconstruction, which is based on a temporal sequence of 3D scans and images. The acquisition of 3D scans can come from depth camera, LiDAR, and so on. These sensors are becoming increasingly affordable and widely used for many applications.

Among the three-dimensional (3D) sensing devices, LiDAR (Lighting Detection and Ranging) holds a high measurement accuracy and higher resolution of angles, distance, and speed. Researchers proposed many methods to achieve precise surface reconstruction for LiDAR point cloud. Verma et al. [1], Zhou et al. [2] and Poullis et al. [3] created 3D scenes from LiDAR data. In these methods, noise was removed by classification. Individual building patches and ground points were separated by segmentation, and mesh models were generated from building patches. These approaches were not generalized well to handle objects with arbitrary shapes, since they relied on predefined patterns. Hornung et al. [4] proposed OctoMap which used octree data structure to realize memory-efficient. However, OctoMap lacked details near the surface and didn’t have an arbitrary resolution. In addition to considering accuracy, these 3D reconstruction methods [1,2,3,4,5,6,7] based on LiDAR were almost off-line, because LiDAR sensors collect ten thousand of points per frame and they will collect a huge number of points just in several minutes. However, online reconstruction based on the sparse LiDAR data is more beneficial for real-time applications. Although Lovi et al. [8] and Hoppe et al. [9] realized real-time incremental reconstruction, these sparse reconstruction methods cannot provide a high precision map. Therefore, an efficient incremental reconstruction method is the key to exploit the high accuracy of LiDAR and avoid the problem of huge amount of data accumulation.

In addition to the 3D reconstruction, extraction and fusion of semantic information is also a very necessary part. In semantic segmentation, breakthrough progress has first been made on image semantic segmentation with CNN based methods in recent years. Long et al. [10] greatly improved the segmentation result by proposing a fully convolutional network, which replaced fully connected layers with convolutional layers and added skip layers to avoid excessive loss of spatial information. On this basis, Zhao et al. [11], Yang et al. [12], Chen et al. [13] and Chen et al. [14] utilized hierarchical context information extraction modules in order to further improve the accuracy. For real-time semantic segmentation task, Zhao et al. [15] and Yu et al. [16] combined image pyramid mechanism with fully convolutional network (FCN), which acquired a good trade-off between accuracy and inference time.

Two-dimensional (2D) segmentation methods are mainly for images. In order to obtain the results of 3D point cloud semantic segmentation, 3D and four-dimensional (4D) semantic segmentation networks have also been extensively studied. Qi et al. [17] and Tchapmi et al. [18] directly convolved on a dense representation. It suffered from high memory consumption. Graham et al. [19] and B et al. [20] used sparse 3D-convolution for more reasonable cost. Alonso et al. [21] and Xu et al. [22] projected the point cloud into 2D space, and used 2D segmentation methods to obtain semantic information. Some methods like PointNet-based [23,24], tree-based [25] and graph-based [26,27] were also very popular, which straightly processed point cloud. Choy et al. [28] used high-dimensional sparse convolution to directly convolve 3D video sequences to propose a 4D perception network, which was more robust to data noise than 3D methods. Although these networks achieve remarkable results on 3D semantic segmentation, they limit the input data to the entire data sequence or the stitched data and need too much inference cost, which make them not suitable to be used in real-time semantic mapping.

Apart from using 3D or 4D semantic segmentation networks to obtain 3D semantic information, these methods [29,30,31,32,33,34,35,36] chose to extract the initial semantic information through the CNN-based 2D network first, and then projected it into the 3D space, which is more efficient and more suitable for real-time applications. Because of the inconsistency of image results over time, an incremental semantic fusion part is necessary for a consistent understanding of 3D space. Xiang et al. [29] stored probability vector of the semantic label space in addition to the TSDF value for each voxel in KinectFusion [37], then a running average method was used for both the TSDF and the probability vector to reduce noise in the fusing process. In [38], running average method was also utilized, but the fusion weight of each voxel for new frame was not uniformly set to 1. Instead, a per-class fixed weight was adopted to resolve the fusion problem of dynamic scenes. In order to reduce resource consumption, Pham et al. [30] and Cavallari et al. [31] stored the confidence vector of label category for each voxel and only current best label and its confidence were output from 2D network. In the processes of updating, the confidence level of the current predicted category was increased while others were punished. Approaches such as [32,33,34,36] utilized the same method as SLAM correspondence for key frame data processing, that is, the recursive Bayesian rule to update the prediction result of each voxel. Although the methods mentioned above can achieve real-time performance, these methods can only exploit historical states, and cannot make full use of the 2D image features and spatial information.

In this paper, a real-time TSDF (Truncated Signed Distance Field)-based 3D semantic reconstruction framework for LiDAR point cloud is presented. We use a line-of-sight algorithm to assign the TSDF value and an attention-based spatial-temporal feature fusion mechanism in order to fuse semantic 3D map during data collection processes. By fusing data over time, our method achieves high accuracy of 3D surface estimation and semantic segmentation both in virtual datasets and real datasets. Especially in semantic segmentation, compared with probability-based method, our method gets 13.3 percent mIoU improvement in Apollo dataset, which is a more challenging real dataset. Besides, our method supports an incremental update of voxels and parallel computation is implemented to achieve real-time performance. This paper makes main contributions as follows,

- We propose a line-of-sight algorithm to update the implicit surface incrementally and reduce noise considering LiDAR characteristics.
- We present a novel self-attention based spatial-temporal feature fusion method for high accuracy of semantic segmentation.
- We present a real-time SDF based 3D semantic reconstruction framework for LiDAR point cloud. Compared with other methods, our framework can achieve smoother 3D surface estimation, higher accuracy of semantic segmentation and real time performance.

## 2. Materials and Methods

**Figure 1:**
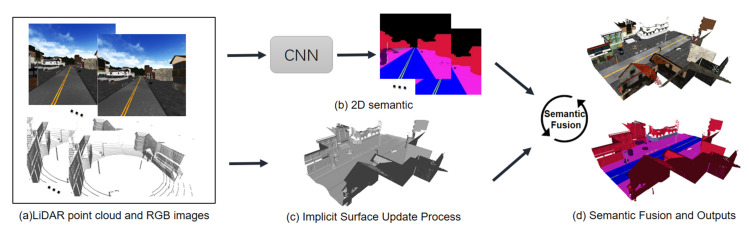
Overview of our pipeline. (**a**) multi-frame Lighting Detection and Ranging (LiDAR) point clouds and RGB images as input; (**b**) two-dimensional (2D) semantic segmentation network; (**c**) the implicit surface update process; and, (**d**) semantic fusion module and 3D semantic mapping results.

The pipeline of our system is shown in Figure 1. The input of our system is the LiDAR point clouds and RGB images. Through the registration algorithm [39], a pose can be estimated and used to map and reconstruct. After obtaining the pose information, voxels will only be created if they are near the surface and within the volume range. Our line-of-sight algorithm updates them incrementally. At the same time, the 2D semantic segmentation network [15] will process the current frame data and output the semantic segmentation results. At last, the semantic fusion module will fuse the semantic results over time into reconstructed 3D map and output 3D semantic map.

### 2.1. 3d Surface Reconstruction

**Figure 2:**
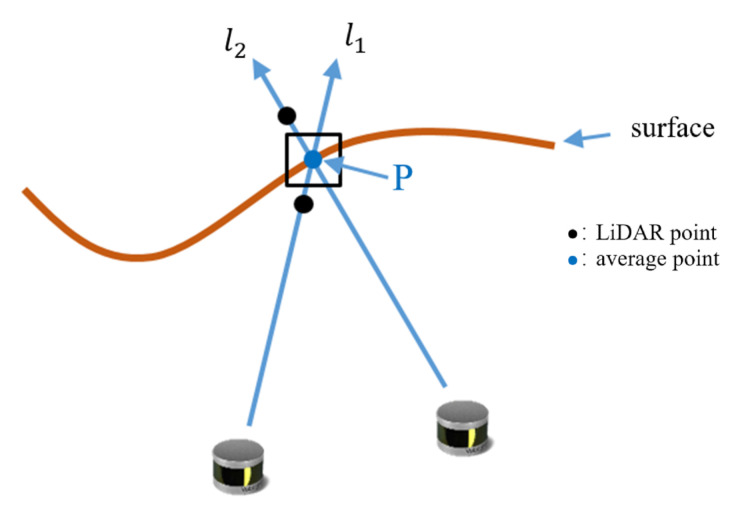
Implicit surface reconstruction. we use TSDF weighted moving average computation. $l_{1}$ and $l_{2}$ are different lines of sight. The first update $l_{1}$ and the next update $l_{2}$ combine to get an average surface point, which is close to the real point.

Current methods [37,40] of integrating depth sensor data into a TSDF are based on depth images. When it comes to LiDAR, it will be different. What LiDAR collects is unorganized point cloud data and we cannot directly use the projection method to update its implicit surface. We propose a voxel based line-of-sight update algorithm, as shown in Figure 2, to solve the problem. The line of sight is gotten from current sensor posture and LiDAR points *P* in world coordinate. We get the voxels that the line of sight cross through, update the associated voxel values, fuse them over time, and get the continuous implicit surface of an object.

The input to our algorithm is an unorganized 3D point cloud set $P_{k}^{L}=\left\{p_{k,1}^{L},p_{k,2}^{L},\cdots ,p_{k,n}^{L}\right\}$ in LiDAR coordinate system {L}, $k\in Z^{+}$, where *k* represents LiDAR frame number, $p_{k,i}^{L}\in R^{3}$ indicates *i*th point in *k*th frame. LiDAR *k*th frame’s pose in world coordinate system is $H_{k}$.

The key idea of our implicit surface update method is to generate the line of sight $\bar{o_{k}p_{k,i}}$, where $o_{k}$ is the sensor origin and $p_{k,i}^{L}$ is the current LiDAR point, and then to find the relevant voxels $X_{p_{k,i}}$ from the line of sight. We transform the point cloud into world coordinate system while using *k*th posture, which contains a 3×3 rotation matrix $R_{k}$ and 3×1 translation vector $T_{k}$.

(1) $$p_{k,i}=\begin{array}{cc} {}[R_{k} & T_{k}]p_{k,i}^{L} \end{array}$$

$o_{k}$ is equal to $T_{k}$ and the line of sight is a three-dimensional vector, as shown in Equation (2).

(2) $$\vec{o_{k}p_{k,i}}=p_{k,i}-o_{k,i}$$

We use the line of sight to sweep relevant voxels in space and update their TSDF values and weights to avoid missing voxels when searching the surrounding voxels of each line of sight. We find maximal axis and take the maximal axis as standard and normalize other two directions to get the normalized direction vector $\hat{\vec{op_{k,i}}}$, as shown in Equation (3).

(3) $$\hat{\vec{o_{k}p_{k,i}}}=\frac{\vec{op_{k,i}}}{max({\vec{op_{k,i}}}_{x},{\vec{op_{k,i}}}_{y},{\vec{op_{k,i}}}_{z})}$$

We then use the line normalized direction vector $\hat{v}$ to find related points $P_{\bar{op_{k,i}}}$ in the front of and behind the original point $O_{k,i}$ respectively, as shown in Equation (4).

(4) $$P_{\bar{op_{k,i}}}=O_{k,i}+m\cdot \hat{\vec{op_{k,i}}}$$

where *m* is a parameter, $m=k_{step}*L_{voxel}$. In our system, $k_{step}$ is set to integers, $k_{step}\in \left\{-5,-4,...,5\right\}$, $L_{voxel}$ is the voxel size. Every time that $k_{step}$ is determined, a related point can be determined. Thus, the related points of the original point can be obtained.

LiDAR probably collects points from a long distance, which contain more noise when compared with close points. We use a dynamic weight to account for noise data far from the sensor. As shown in Equation (5), *a* is a parameter that is related to the size of reconstruction scene.

(5) $$w_{v_{k,i}}=\frac{a}{a+\left|\right|\vec{o_{k}p_{k,i}}\left|\right|}$$

TSDF is updated as Equations (6) and (7).

(6) $$W_{k}\left(x_{k,i}\right)=max\left(W_{k-1}\left(x_{k,i}\right)+w_{i}\left(x_{k,i}\right),W_{max}\right)$$

(7) $$D_{k}\left(x_{k,i}\right)=\frac{W_{k-1}\left(x_{k,i}\right)D_{k-1}\left(x_{k,i}\right)+w_{k}\left(x_{k,i}\right)d_{k}\left(x_{k,i}\right)}{W_{k}\left(x_{k,i}\right)+w_{k}\left(x_{k,i}\right)}$$

where $D_{k-1}\left(x_{k,i}\right)$, $W_{k-1}\left(x_{k,i}\right)$ are the TSDF values and their weights of all voxels in *k*-1 frame, as shown in Equation (8).

(8) $$\begin{array}{cc} D_{k-1}\left(x_{k,i}\right) & =\frac{\sum w_{k-1}\left(x_{k,i}\right)d_{k-1}\left(x_{k,i}\right)}{\sum d_{k-1}\left(x_{k,i}\right)}\\ W_{k-1}\left(x_{k,i}\right) & =\sum w_{k-1}\left(x_{k,i}\right) \end{array}$$

Voxel’s signed distance function is $d_{1}\left(x_{k,i}\right)$, $d_{2}\left(x_{k,i}\right)$, ⋯$d_{n}\left(x_{k,i}\right)$ with corresponding weight $w_{1}\left(x_{k,i}\right)$, $w_{2}\left(x_{k,i}\right)$, ⋯$w_{n}\left(x_{k,i}\right)$.

For parallelization, we use a spatial hashing-based data structure in order to manage the space, which allows for us to insert and update voxels in parallel. A memory pool is also created to reduce fragmentation of memory and improve efficiency. We only insert voxels near the zero isosurface and do not waste memory on empty space.

### 2.2. Semantic Fusion

The input of our system is LiDAR point clouds and RGB images. Each LiDAR point can be indexed to a voxel and we only update these related voxels. Firstly, the incremental reconstruction result provides voxels’ normal that is embedded with position of the sensor as the input of our Observation Adaptive Network (OAN) to provide the observation effectiveness information. Secondly, through the 2D semantic segmentation network, image feature is extracted and LiDAR point cloud is projected to obtain its corresponding image feature. The image feature of current voxel is also the input of our OAN, and it is used together with voxel’s normal and position of the sensor to update current voxel’s state. Finally, we use Attention Based Spatial Fusion Network (ABSFN) to fuse voxels’ state within a limited range of adjacent space to obtain current voxel’s semantic label. All of the modules will fuse the semantic results over time into reconstructed 3D map and output 3D semantic map.

#### 2.2.1. Network’s Input

Image Feature. Our Feature Fusion Network requires voxels’ corresponding image feature $R_{i}^{k}$, where *i* represents *i*th voxel and *k* represents *k*th frame. To achieve this, we first train a 2D real-time segmentation network [15] in order to extract dense image feature. Then the LiDAR points are projected onto the camera plane to get semantic feature of each voxel according to the corresponding pixel. In projection process, some voxels may lie out of the camera plane and these voxels are not reconstructed.

Normal. Our system performs 3D reconstruction incrementally that is based on SDF (Signed Distance Field) algorithm. By calculating the gradient of signed distance field, normal of the *i*th voxel in *k*th frame can be achieved, which is denoted by $N_{i}^{k}$.

Position of the sensor. In order to represent the validity of the observation from a geometric perspective, we also need current frame sensor position, which is equal to $o_{k}$ in Section 2.1. We use $L_{i}^{k}$ in order to represent it in this section, where *i* represents *i*th voxel and *k* represents *k*th frame.

#### 2.2.2. Observation Adaptive Network.

**Figure 3:**
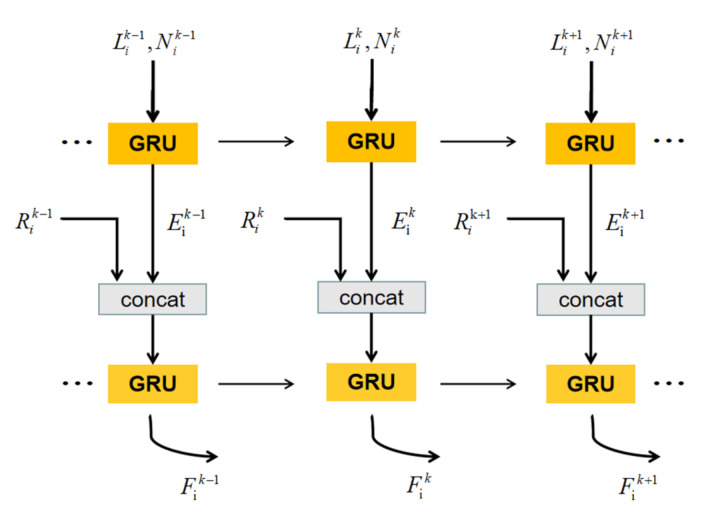
The structure of Observation Adaptive Network (OAN).

Multi-view observation is considered to be beneficial for robust scene understanding. However, the changes of observation are not uniform in the process of data collection. Especially when sensors stop, a large amount of data are collected from the same pose, which is redundant. Therefore, we design the OAN to evaluate observation effectiveness. The structure of OAN is shown as Figure 3. We assume there are two main factors related to the effectiveness of observations. Firstly, the location of observation $L_{i}^{k}$ in the local coordinate system centered on current voxel. Secondly, the normal of current voxel $N_{i}^{k}$. The combination of normal and position can represent the validity of the observation from a geometric perspective. When the observation degree is close to 90 degrees from normal, the observation is not reliable. When the observation degree is close to 0 degree, the observation is reliable. Consequently, we utilize GRU to achieve the observation state $E_{i}^{k}$, which uses voxel’s normal and sensor’s local position as input.

(9) $$E_{i}^{k}=GRU\left(L_{i}^{k},N_{i}^{k},E_{i}^{k-1}\right)$$

where $E_{i}^{k}$ implicitly contains the information of observation effectiveness.

Subsequently, image feature $R_{i}^{k}$ achieved from 2D images is concat with $E_{i}^{k}$ as the input of next layer of GRU.

(10) $$F_{i}^{k}=GRU\left(concate\left(R_{i}^{k},E_{i}^{k}\right),F_{i}^{k-1}\right)$$

$F_{i}^{k}$ is the hidden layer state of GRU that will be saved in each voxel. When the specific category of voxel needs to be calculated, ABSFN will obtain the status from the relevant voxel to obtain the final result.

#### 2.2.3. Attention Based Spatial Fusion Network

Output from the OAN does not exploit spatial neighborhood information, which is helpful to further improve the accuracy of semantic segmentation. A direct method to exploit the spatial neighborhood information is performing 3D convolution on sparse 3D data. However, this method is inefficient, especially when voxels maintain a high dimensional feature. In addition, the 3D structure changes over time, which makes it hard for fixed grid network to learn. Therefore, we use the self-attention mechanism to explicitly measure the correlation between the current voxel and its adjacent voxels. The normalized sum unit makes the network more robust to the change of 3D structure. Conclusively, we propose ABSFN.

Looking Up Neighborhood. Our 3D semantic map uses a spatial hashing-based data structure for efficient space management. When looking up neighborhood voxels, the searching range is limited to a cube that is centered at current voxel. The side length of the cube is *s* voxels. For each grid’s location in the cube, we judge whether there exists the neighborhood voxel. If existing, the voxel will be added to neighborhood list $H_{i}^{k}=\left\{h_{i,1}^{k},h_{i,2}^{k},...,h_{i,j}^{k},...h_{i,N}^{k}\right\}$, where *j* represents *j*th neighborhood voxel and *N* represents the number of neighborhood voxel.

**Figure 4:**
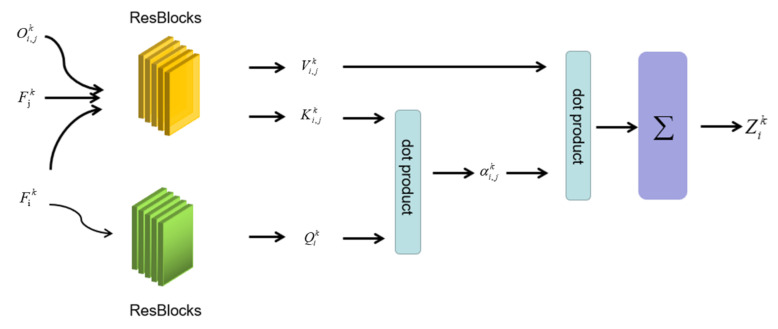
The structure of Attention Based Spatial Fusion Network (ABSFN).

Spatial Self-attention. We assume there are two main elements in our system determining relevance between current voxel and its neighborhood. One is the hidden state stored in each voxel $F_{i}^{k}$, the other is the offset of neighborhood voxels to the current voxel, denoted as $O_{i,j}^{k}$, which means that the offset between *i*th voxel and *j*th voxel in *k*th frame, and the hidden states stored in *j*th voxels denoted as $F_{j}^{k}$. When considering the feature space inconsistency between $O_{i,j}^{k}$ and $F_{i}^{k}$, we design a light-weight encoder to implement space transformation for a better weight measurement. The structure of ABSFN is shown as Figure 4. The output of OAN $F_{i}^{k}$ is embedded with offset $O_{i,j}^{k}$ and split into two branches: one only contains the target voxel’s information to generate $Q_{i}^{k}$, the other, which contains all of the neighborhood voxels, generates weighted feature dictionary, denoting as $K_{i,j}^{k}$ and $V_{i,j}^{k}$, according to the state of voxels themselves. In relevance calculation, we choose dot product to reduce computational complexity. Equations are shown, as below.

(11) $$\begin{array}{c} K_{i,j}^{k},V_{i,j}^{k}=f\left(F_{i}^{k},O_{i,j}^{k},F_{j}^{k}\right) \end{array}$$

(12) $$\begin{array}{c} Q_{i}^{k}=g\left(F_{i}^{k}\right) \end{array}$$

(13) $$\begin{array}{cc} \; & Z_{i}^{k}=\sum_{j=1}^{N}\alpha_{i,j}^{k}*V_{i,j}^{k} \end{array}$$

$Z_{i}^{k}$ is the fusion result of $V_{i,j}^{k}$. We use ResBlocks to train the function of *f* and *g*. The coefficient $\alpha_{i,j}^{k}$ is calculated by feature vector of the target voxel and *j*th neighborhood voxel as the following equation.

(14) $$\begin{array}{cc} \; & \alpha_{i,j}^{k}=Q_{i}^{k}{\left(K_{i,j}^{k}\right)}^{T} \end{array}$$

(15) $$\begin{array}{cc} \; & \alpha_{i,j}^{k}=\frac{\alpha_{i,j}^{k}}{\sum_{j=1}^{N}\alpha_{i,j}^{k}} \end{array}$$

## 3. Results and Discussion

### 3.1. Dataset

We verify the effectiveness of our method for both 3D semantic segmentation and reconstruction in three datasets.

CARLA Dataset. We generate a virtual dataset with CARLA [41] simulation platform, which is built for realism in the rendering and physical simulation and allows for flexible configuration of the agent’s sensor suite. In our experiment, we set cameras with 1024×1024 resolution to obtain RGB and semantic segmentation images. A virtual 64-line 10 Hz LiDAR sensor is also added to obtain point cloud. With sensors attached to a auto-piloted car in the virtual city, we simulate and collect sensor data with corresponding poses at 10 fps. Totally, 900 frames data are generated and every 10th frame of the sequence is selected to yield a 90 frames test set.

Apollo Dataset. We also evaluate our approach on Apollo Dataset [42], which is collected from the real environment. The environment is complex and there is more noise. For real-world large-scale scenes mapping, it is a challenging dataset. We use the Record005 sequence data of Road02 portion.

VLP-16 Dataset. The VLP-16 dataset is collected by ourselves. Our system’s operating frequency is 10 Hz, which includes a VLP-16 LiDAR, two cameras, and a laptop.

### 3.2. Implementation Details

Our experiments were performed on a computer with Intel Core I7 and NVIDA GeForce GTX 1080Ti, and we operated our algorithm by c++ and pytorch.

The input of our method is the LiDAR point clouds and RGB images. Point cloud is transferred into the 3D reconstruction module to get 3D map. In the process of 3D surface reconstruction, we set the value of *a* to 5 empirically, and we set voxel size to 0.1 m to obtain a balance between speed and quality.

RGB images are processed by ICNET [15] to get 2D image feature. For searching neighborhood in ABSFN, we set the value of *s* to 100 and the value of *N* to 25. And we used Adam optimizer to train our semantic fusion network with an initial learning rate of 0.001 and a decay of 0.00001 every epoch. For the optimization, we used the cross-entropy loss function.

**Figure 5:**
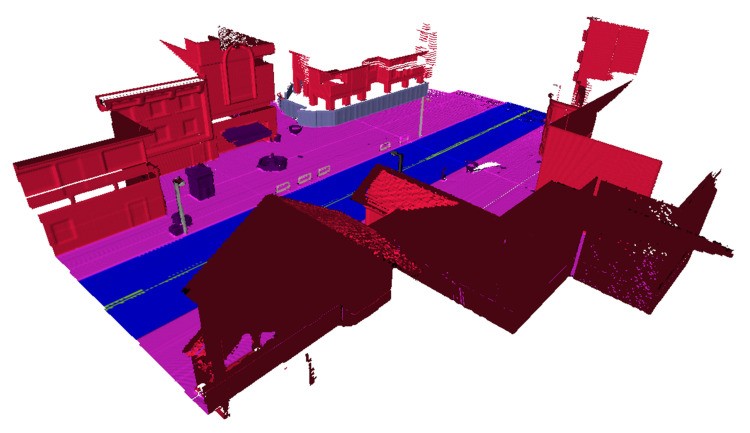
The result of three-dimensional (3D) semantic map. Different colors represent different categories.

The final prediction results of our framework are rendered from 3D semantic map. Figure 5 shows the result by rendering the output of the 3D semantic reconstruction framework.

### 3.3. 3D Reconstruction Results

#### 3.3.1. Qualitative Results

**Figure 6:**
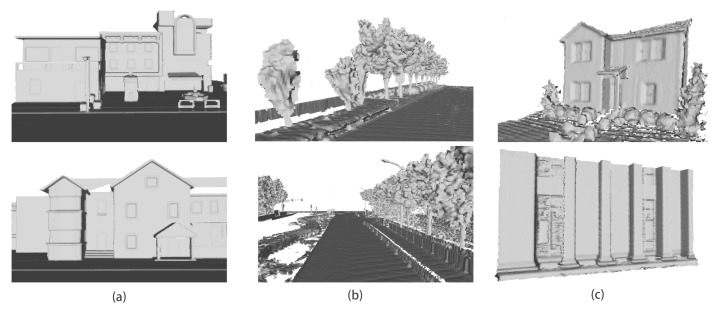
Surface reconstruction results. Each column corresponds to different datasets. (**a**) is from CARLA simulator [41]. (**b**) is from Apollo dataset [42]. (**c**) is the VLP-16 dataset, which is collected from our system.

We show some qualitative results of our reconstruction approach and our method achieves good results on both virtual dataset and real datasets. As shown in Figure 6, our approach not only obtains good results on large objects, such as roads and buildings but also has the ability to accurately recover small object such as ostreet lights, boxes and so on. These results prove that the line-of-sight algorithm is very effective on LiDAR point cloud.

#### 3.3.2. Performance Comparison

**Figure 7:**
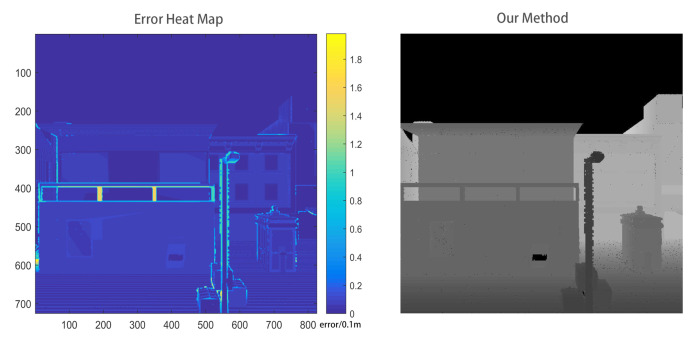
Reconstruction error of our method.

When considering that there is no ground truth in Apollo dataset and VLP-16 dataset, we only reconstruct a scene in CARLA simulator as shown in Figure 7 to evaluate the errors introduced by line-of-sight algorithm. The error of 3D reconstruction mainly appears at the edge of small objects, such as railing and street lights. This is because the incremental reconstruction process will magnify the reconstruction error that is caused by the pose estimation error. However, the overall error value is 0.019m in average.

As the approach of Sengupta et al. [43], we render depth images from our reconstructed 3D model. Error heat maps can be calculated by comparing them with the ground truth. We think one pixel is accurate if the error is less than a fixed threshold and we can get the accuracy of an image. When the depth threshold is 0.2 m and 0.1 m, our approach achieves 94.87% and 86.91% accuracy, respectively, on average.

**Figure 8:**
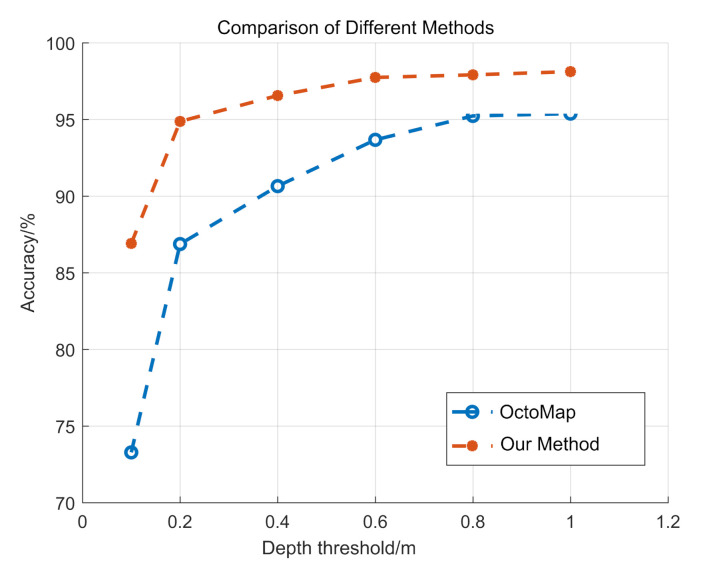
Qualitative comparison of reconstruction result between our methods and OctoMap [4] under different depth threshold.

We compare our approach with OctoMap [4]. OctoMap uses a discrete cut-off probability and its precision is limited by the minimum voxel size. When compared with OctoMap, one advantage of our method is that we can reach the subvoxel precision. We use the same accuracy evaluation method. When the depth threshold is 0.2 m and 0.1 m, OctoMap achieves 86.85% and 73.28% accuracy, respectively, on average. Our approach outperforms OctoMap in all cases, as shown in Figure 8.

### 3.4. Semantic Mapping Results

We quantitatively evaluate the accuracy of our semantic reconstruction result on our simulated CARLA dataset and Apollo dataset. The prediction results of our fusion system are rendered from 3D semantic map. We use pixel-wise interaction over reunion (IoU) as our metric. For a fair comparison, only pixels that were included in rendered map are calculated.

**Figure 9:**
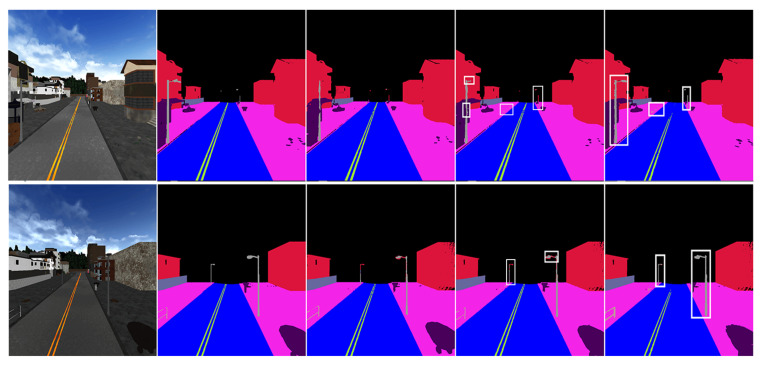
Visualization of CARLA dataset. From the left, RGB images, ground truth, ICNET [15], ICNET [15] +OAN, ICNET [15] + OAN + ABSFN. Different colors represent different categories.

We conduct two experiments to prove the effectiveness of our method. The first comparative experiment conducted on our simulated CARLA dataset shows the effectiveness of OAN and ABSFN. The results are summarized in Table 1 and visualized comparisons are illustrated in Figure 9. We first observe that our OAN utilizing point cloud data achieves a slight improvement of IoU on poles and fence class against [15]. Because our method evaluates the validity of observation, our approach of adaptive observation with attention-based spatial fusion obtains better performance upon all classes except fences and achieves an improvement of 22.01% over [15] on mIoU, owing to the advantage of geometry information. As we can see in Figure 9, many blurred boundaries are eliminated via attention-based spatial fusion approach and the outlines of thin objects improve a lot. The above results demonstrate the effectiveness of our semantic fusion system and the ability to reconstruct a highly precise semantic map. In order to better illustrate our conclusions, we also conduct corresponding experiments on the Apollo dataset, and outline the results in Table 2.

**Figure 10:**
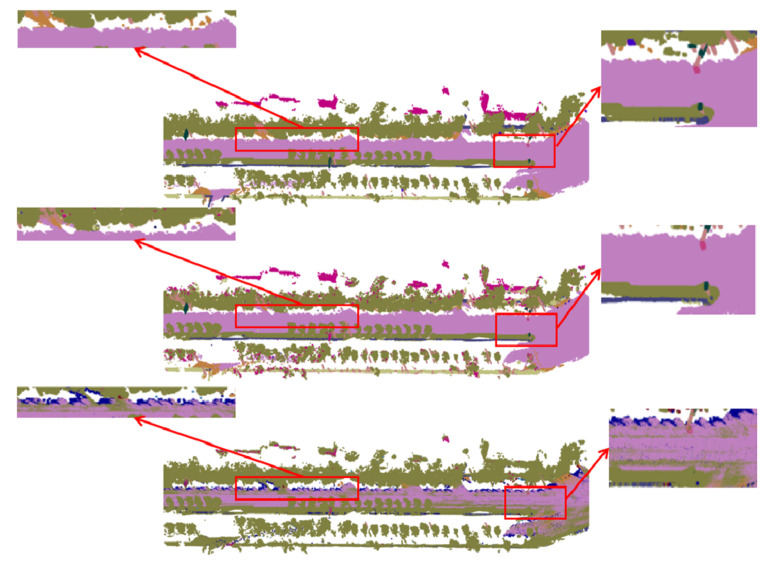
Visualization of Apollo dataset. From the top, ground truth, Ours, ICNET [15]. Our method is significantly better than the ICNET [15] in spatial consistency.

**Figure 11:**
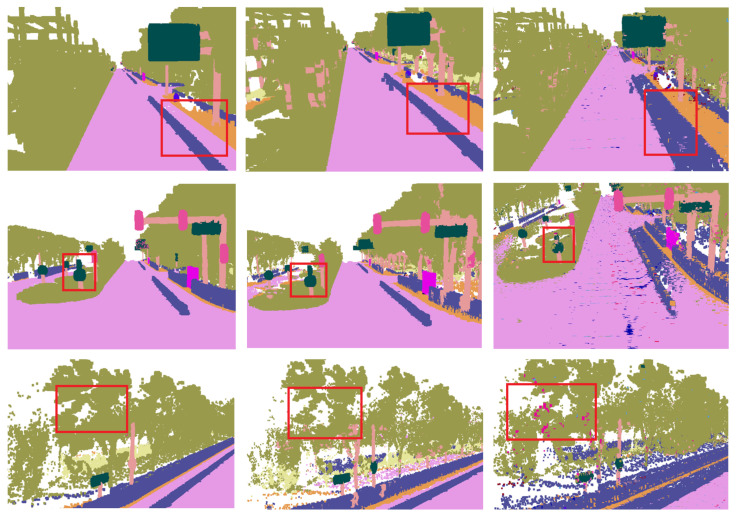
Visualization of Apollo dataset in detail. From the left, ground truth, Ours, [32]. The accuracy of our method in the red boxes is significantly higher than [32].

We compare our method with other related methods. Because our framework hopes to be able to process each frame data in real time. Therefore, we choose some methods that can extract the semantic information of each frame in real time for fair comparison. On the other hand, fusion is the focus of our framework, many 2D or 3D semantic segmentation networks with high performance can be the input of the fusion in our framework, so we only compare our methods with similar fusion methods and basic 2D or 3D semantic segmentation methods. Finally, we compare our method with the ICNET [15], the Pointnet [23], and [32]. The ICNET [15] gets the semantic segmentation results through the CNN-based 2D network, and then we project the semantic segmentation results into the 3D space without extracting spatial and temporal information. The Pointnet [23] considers 3D spatial information but does not consider temporal information. Li et al. [32] considers temporal information but does not consider spatial information and 2D images features. From the quantitative results, our method has a greater improvement in accuracy. Table 3 presents the results obtained from the Apollo dataset which is a more challenging real dataset. It can be seen from the Table 3 that the semantic estimation that is based on probability fusion [32] has a certain improvement in accuracy compared to the semantic segmentation directly on the point cloud [23] or the projection method without fusion [15]. What is more, when compared with the probability-based method, our method gets a 13.3 percent mIoU improvement. Because our method takes texture and context information into account in fusion. The visualized comparisons are illustrated in Figure 10 and Figure 11. Figure 10 is the visualization result of a sequence. From the top are ground truth, the results of our method and the results of the ICNET [15]. As we can see from the red boxes in Figure 10, there is a lot of high-frequency noise in the result of ICNET [15]. These high-frequency noise can also be regarded as discontinuous in 3D space. However, in the results of our method, these high-frequency noise is eliminated. Figure 11 shows the details of 3D semantic segmentation on the Apollo dataset. From the left are ground truth, the results of our method and results of [32]. In the red boxes of Figure 11, the accuracy of classification is well improved in our method. This shows that our method integrates spatial geometric information and temporal information very well.

### 3.5. Real-Time Performance

**Figure 12:**
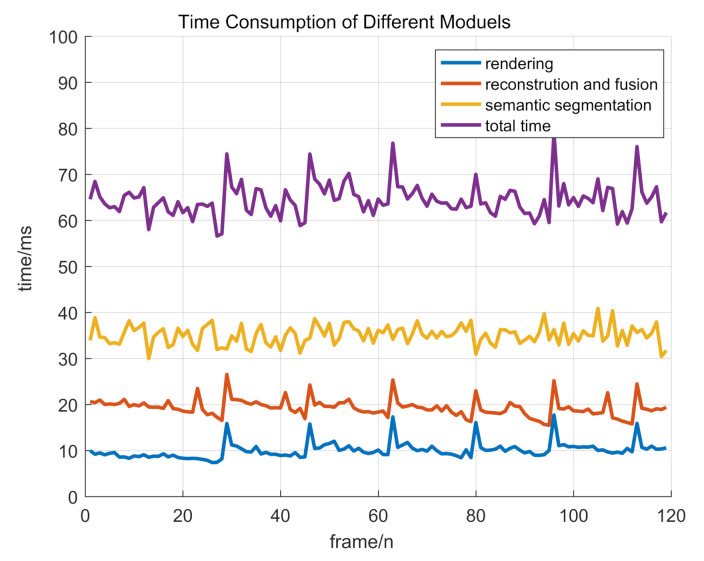
Time Consumption of different modules, including rendering, semantic segmentation, reconstruction, and fusion. The total time is about 65 ms in average.

Real-time performance means that all of our software modules should be completed before the next frame’s data coming. In our system, LiDAR’s operating frequency is 10 Hz and we need to process data within 100 ms per frame. VLP-16 LiDAR collects about 20k points per frame. Accordingly, we fix the points number per frame to 20k in Table 4. According to the experiment in Table 4, we set the voxel size to 0.1 m for the balance between speed and quality. As shown in Table 5, our system is robust when the points number changes. greenSetting the voxel size and points number to 0.1 m and 20k, the total time per frame is about 65 ms in average. Although there are a lot of related works on reconstruction or semantic segmentation, there are few works on both of them. Therefore, for a fair compassion, we only compare our method with [32], which conducts 3D reconstruction and semantic segmentation at the same time. For [32], with the same voxel size and points number, the total time per frame is about 266 ms in average. Therefore, our system is up to four times faster than [32]. In our system, time spending on the semantic segmentation, reconstruction, and rendering is about 35 ms, 20 ms, and 10 ms on average, as shown in Figure 12. Finally, our system consumes less than 0.1 s per frame and achieves real-time performance.

## 4. Conclusions

In this paper, we propose a real-time framework in order to generate a 3D semantic map in outdoor environment by combining LiDAR point clouds and RGB images. A line-of-sight algorithm is used to reconstruct smooth surface from LiDAR data with large noise. With respect to semantic mapping, an online attention-based spatial and temporal feature fusion network is proposed to incrementally update the semantic information. Our method provides a real-time performance to meet robotics needs and it achieves high accuracy of 3D surface estimation and semantic segmentation. When compared with the state-of-art mapping methods, our method has a great advantage in terms of quality and speed, which meets the needs of robotic mapping and navigation.

## Figures and Tables

**Table 1:** Comparison on CARLA dataset. The bold fonts indicate the best results.

Method	Buildings	Fences	Poles	Roadlines	Roads	Sidewalks	Vegetation	Vehicles	Walls	Others	mIoU
ICNET [15]	94.88	38.50	61.62	66.94	96.64	94.71	69.81	92.77	83.41	61.67	66.43
ICNET [15] + OAN	95.91	**50.86**	80.32	83.24	98.77	95.89	76.13	99.11	87.47	69.08	83.68
ICNet [15] + OAN + ABSFN	**96.35**	50.60	**81.20**	**84.92**	**99.05**	**97.24**	**76.93**	**99.64**	**88.01**	**70.29**	**84.42**

**Table 2:** Comparison on Apollo dataset. The bold fonts indicate the best results.

Method	Road	Sidewalk	Traffic Cone	Road Pile	Fence	Traffic Light	Pole	Traffic Sign	Wall	Dustbin	Billboard	Building	Vegetations	mIoU
ICNET [15]	72.39	37.02	0.14	1.35	46.21	29.64	18.61	28.52	3.94	4.16	8.01	28.96	84.17	27.82
ICNET [15] + OAN	97.02	53.54	0.27	2.64	57.63	32.64	24.68	53.56	32.90	4.39	41.90	**47.33**	91.86	41.57
ICNet [15] + OAN + ABSFN	**97.62**	**58.77**	**8.71**	**46.04**	**65.21**	**51.56**	**28.55**	**77.54**	**22.23**	**30.20**	**83.82**	44.82	**91.88**	**54.38**

**Table 3:** Segmentation results of different methods on the Apollo dataset. The bold fonts indicate the best results.

Method	mIoU
ICNET [15]	27.8
PointNet [23]	30.8
probability fusion [32]	41.7
ours	**54.4**

**Table 4:** 20k points per frame.

Voxel Size/m	Time/ms
0.2	42
0.15	52
0.10	65
0.08	88
0.06	122
0.04	136
0.02	153

**Table 5:** Voxel size is 0.1 m.

Points Number (per Frame)	Time/ms
5k	37
10k	45
20k	65
40k	113
60k	152
80k	213
100k	265
